# Targeting Microglia in Alzheimer’s Disease: From Molecular Mechanisms to Potential Therapeutic Targets for Small Molecules

**DOI:** 10.3390/molecules27134124

**Published:** 2022-06-27

**Authors:** Ziyad M. Althafar

**Affiliations:** Department of Medical Laboratories Sciences, College of Applied Medical Sciences in Alquwayiyah, Shaqra University, Riyadh 11961, Saudi Arabia; zalthafar@gmail.com

**Keywords:** Alzheimer’s disease, pathogenesis, microglia, amyloid-beta, tau, neuroinflammation

## Abstract

Alzheimer’s disease (AD) is a common, progressive, and devastating neurodegenerative disorder that mainly affects the elderly. Microglial dysregulation, amyloid-beta (Aβ) plaques, and intracellular neurofibrillary tangles play crucial roles in the pathogenesis of AD. In the brain, microglia play roles as immune cells to provide protection against virus injuries and diseases. They have significant contributions in the development of the brain, cognition, homeostasis of the brain, and plasticity. Multiple studies have confirmed that uncontrolled microglial function can result in impaired microglial mitophagy, induced Aβ accumulation and tau pathology, and a chronic neuroinflammatory environment. In the brain, most of the genes that are associated with AD risk are highly expressed by microglia. Although it was initially regarded that microglia reaction is incidental and induced by dystrophic neurites and Aβ plaques. Nonetheless, it has been reported by genome-wide association studies that most of the risk loci for AD are located in genes that are occasionally uniquely and highly expressed in microglia. This finding further suggests that microglia play significant roles in early AD stages and they be targeted for the development of novel therapeutics. In this review, we have summarized the molecular pathogenesis of AD, microglial activities in the adult brain, the role of microglia in the aging brain, and the role of microglia in AD. We have also particularly focused on the significance of targeting microglia for the treatment of AD.

## 1. Introduction

Alzheimer’s disease (AD) is a complex neurodegenerative disease (ND) and the characteristics of AD include cognitive deficit and memory loss that can eventually disrupt the motor system, visuospatial orientation, speech, and behavior [1,2]. Several neuropathological hallmarks of AD including inflammation, intracellular neurofibrillary tangles (NFTs), and extracellular Aβ deposits have already been well identified [3,4,5]. Tau proteins and deposits of Aβ peptides are detected in different brain areas, which can further lead to microglial activation, mitochondrial dysfunction, synaptic dysfunction, and even neuronal cell death [6,7]. Characteristics of AD-related inflammation include reactive microglia around Aβ plaques, which preserve an inflammatory state via releasing various proinflammatory mediators, which can ultimately lead to neuronal loss. In the case of AD, reactive gliosis histology indicates the aberrant morphology and proliferation of microglia and astrocytes. It has been reported that astrogliosis and microgliosis are common characteristics of numerous NDs with different causes [8,9,10], however it was not certain whether these histopathological alterations reflect an inconsequential, harmful, or beneficial function of glial cells in the neurodegenerative process. Unfortunately, currently available drugs can only provide symptomatic treatment of AD (Table 1), instead of curing or preventing this devastating disease. 

Microglia play role as the resident immune cells of the central nervous system (CNS) [11,12]. Self-renewal and homeostasis of microglia is maintained via various factors, such as macrophage-colony stimulating factor and transforming growth factor signaling [13,14,15]. Microglia play crucial roles in CNS tissue maintenance, pathogen defense, and injury response [16,17]. In addition, they play roles in the developmental sculpting of neural circuits via engulfing and removing unwanted synapses and neurons [18,19]. Microglia have multifaceted roles in the course of AD because of their various phenotypes and a range of activation pathways. After pathological stimulation, extremely branched microglia have the capacity to alter to an amoeboid form [20,21]. In aging brains, microglia have reduced level of branching that decreases their capacity of surveillance, which can further lead to impaired homeostatic functions [22,23,24]. In diseased brains, morphology of microglia differs owing to the spatial location and AD stage. Amyloid plaque-linked microglia also go through dramatic and morphological alterations, while plaque-distant microglia exhibit minor alterations over time [25]. As compared to the brains at earlier AD stages, microglia in Braak stage V–VI brains possess more intense morphological alterations [26]. The progressive variety in microglial structure may take place because of the duration and intensity of the pathological environment [27], however this may also take place because of the differences in responses of microglia to different stimuli including tau or Aβ aggregation [28]. It has been reported that dystrophic microglia emergence precedes tau pathology development [29,30].

**Figure 1:**
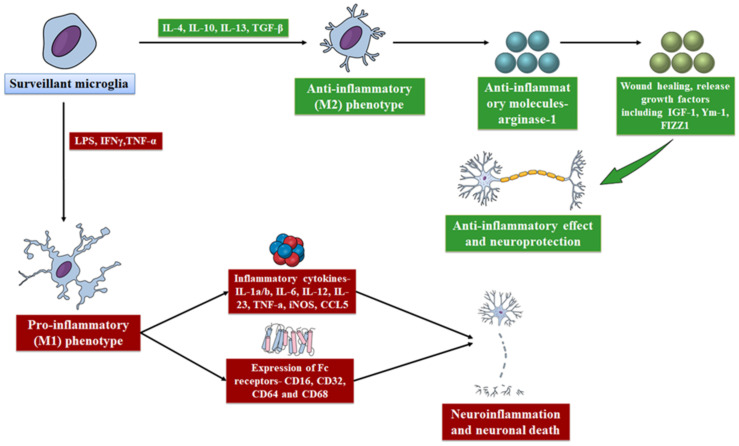
Schematic description of M1 and M2 polarization of microglia and their immunoregulatory functions.

Soluble form of hyperphosphorylated tau may trigger the phenotypic alteration in microglia, which can further cause loss of immunosurveillance activity and mediate AD progression via the formation of NFTs [26]. Collectively, these findings suggest that phenotypic alterations in microglia including behavior, proteomic signatures, and morphology are linked with AD progression [31,32]. It has been revealed that M1 microglia secrete various inflammatory chemokines and cytokines, which can result in neuronal death and inflammation (Figure 1) [33], whereas tissue repair and maintenance are linked with alternative M2 microglial activation [34]. In addition, M2 microglia mediate neuroprotection and anti-inflammatory effects, whereas M1 microglia mediate neurotoxicity and inflammation. Indeed, both of these phenotypes play roles in the NDs, thus microglia have the capacity to play role as a double-edged sword in NDs [35]. Therefore, precise regulation of microglia activation is important for the normal activity of microglia to prevent NDs and maintain brain homoeostasis [36].

In AD mice, parabiosis studies showed that microglia are responsible for the elevated level of myeloid cells found in brains containing plaque pathology, along with the negligible impact of infiltrating macrophages [37]. In this review, we have highlighted the molecular pathogenesis of AD, microglial functions in the adult brain, the effect of microglia in the aging brain, and the effect of microglia in AD. Furthermore, we have focused on the importance of targeting microglia for AD treatment. Information for the review was gathered from a variety of sources and databases, including Science Direct, Google Scholar, PubMed, and Scopus. Several keywords were utilized in this study, including Microglia, Alzheimer’s disease, amyloid-beta, brain, Aβ plaques, tau protein, neuroinflammation, therapeutics, CSF1R, immunoreceptors, etc. The data were gathered from manuscripts, theses, books, book chapters, conference proceedings, and other publications published till 2022.

**Table 1:** Currently available therapies for Alzheimer’s disease treatment.

Drug	Approved Indication	Mode of Action	Dose	Titration Scheme	References
Memantine	Moderate-to-severe Alzheimer’s disease (AD)	Non-competitively antagonize N-methyl-D-aspartic acid receptor	5–20 mg/day	Initially 5 mg/day, subsequently increase 5 mg at weekly intervals to a maximum dose of 20 mg/day	[2,38,39,40,41,42,43]
Galantamine	Mild-to-moderate AD	Selectively, reversibly, and competitively suppress AChE	16–24 mg/day	Initially 8 mg once per day for four weeks, subsequently increase to 16 mg once per day for minimum four weeks; maintenance therapy is 16–24 mg once per day	[38,39,42,43,44]
Rivastigmine	Mild-to-moderate AD	Pseudo-selectively and irreversibly suppress butyrylcholinesterase and AChE	1.5–6 mg/day	Initially 1.5 mg two times per day and the dose can be increased up to 1.5 mg two times per day at intervals of minimum two weeks as per the tolerance; the maximum dose is 6 mg two times per day	[38,39,42,43,45]
Donepezil	All stages of AD	Selectively, non-competitively, and reversibly suppress AChE	5–10 mg/day	Initially 5 mg/day; if necessary, the dose can be increased up to 10 mg after 1 month	[38,39,42,43,46,47]

## 2. Molecular Pathogenesis of Alzheimer’s Disease

AD is widely known as a multifactorial and complex ND [48]. Several factors play roles in AD pathogenesis including Aβ generation, hyperphosphorylated tau, neuroinflammation, endoplasmic reticulum stress, aberrant mitochondrial activity, and elevated oxidative stress (OS) [49]. Elevated OS is regarded as one of the key role players in AD pathogenesis [50]. OS can take place owing to the excessive generation of reactive oxygen species (ROS). ROS can be generated in the case of various normal physiological settings (for example- during cellular metabolism in the mitochondria), whereas the excessive level of ROS can be generated during a diseased state [51]. It has been observed that mitochondrial dysfunction or decreased function of various endogenous antioxidants including catalase, glutathione, and superoxide dismutase is responsible for ROS generation [51]. 

Excessive levels of ROS generation can lead to DNA damage, malonaldehyde (MDA) generation, lipid oxidation, and modulation of the peroxy-nitrite (ONOO) generation via controlling the inducible nitric oxide synthetase (iNOS) transcription. Therefore, elevated levels of ONOO, iNOS, and ROS can result in the generation of reactive nitrogen species (RNS), which can ultimately play roles in AD pathogenesis [50]. It has been observed that elevated levels of RNS and ROS can cause activation of glial cells, NLR family pyrin domain containing 3 (NLRP3) inflammasome modulation, initiation of various neuroinflammatory signaling pathways (TLR-4/p38 MAPK/NF-kB), and induce the generation of NFT and Aβ via controlling the Nrf2/JNK/Wnt/GSK-3β pathways [52,53,54]. Interestingly, elevated levels of nitrative stress and OS can react with amyloid precursor protein (APP) and various enzymes that are linked with the Aβ generation and therefore can modulate the deposition and generation of Aβ [55]. 

In a normal brain, Aβ plays several neurophysiological roles and its clearance from the brain takes place via several processes. APP is regarded as the key role player in Aβ generation and clearance. In addition to α, β, and γ secretases, APP is also linked with Aβ homeostasis [56,57]. It is widely known that β and γ secretases are accountable for the Aβ production. On the other hand, low-density lipoprotein receptor-related protein is accountable for Aβ clearance from the brain and transferring it into the systemic circulation, where it gets excreted through hepatic and renal metabolic pathways [58]. Nonetheless, an elevated level of RNS and ROS causes Aβ generation by inducing the catalytic effects of β and γ secretases, while this elevated level limits LPR-caused Aβ excretion and therefore elevates the deposition and generation of Aβ [59].

## 3. Functions of Microglia in Healthy Adult Brain

In an adult brain, microglia exist in a sedentary or resting condition, however in this condition, they keep monitoring the healthy brain for any unwanted situation. It has been observed that during such inspections, microglial processes directly interact with synapses [60]. Furthermore, in a healthy brain, the resting microglial cells reside in strategic areas throughout the spinal cord and brain to identify and fight against infections [61]. In resting conditions, microglia secrete several neurotrophic growth factors to improve neurogenesis and also to mediate the survival of neurons [62]. The resident microglia get induced during brain insults and NDs and further get transformed into reactive or activated microglia. Moreover, during these conditions, microglia secrete numerous reactive free radicals, prostanoids, chemokines, matrix proteins, growth factors, and inflammatory molecules, which further play roles in cell death and neuronal dysfunction or mediate the healing process of injured tissues [63]. Interestingly, the (beneficial or harmful) activity of microglia relies on the injury and extent of related microglial activation. Various studies have reported the capacity of adult brain microglia in re-establishing their normal density if reduced experimentally [15,64]. 

**Figure 2:**
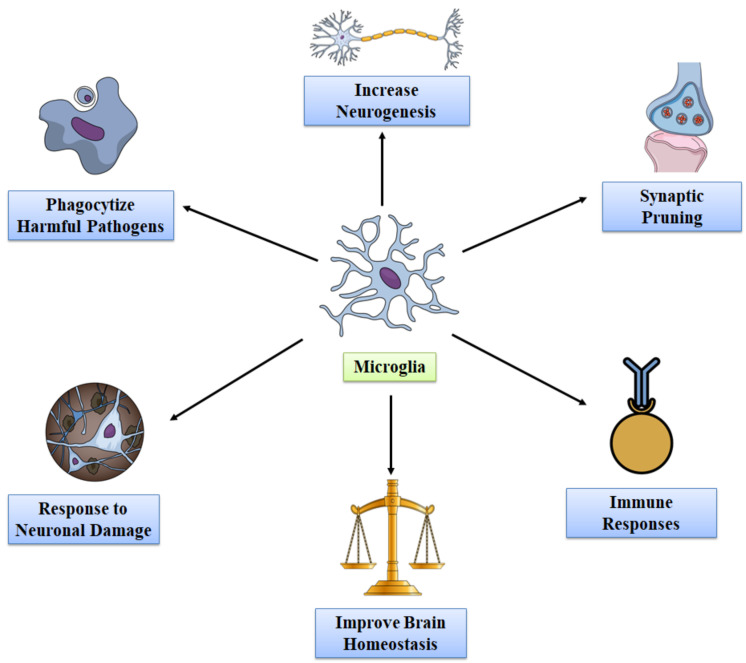
The beneficial effects of microglia in healthy adult brains.

In addition, damaged and/or old microglia get replaced with new healthy microglia during aging and disease conditions. In the adult brain, microglia help in tuning synapse strength and regulating long-term potentiation (LTP), which is accountable for constant long-term neural networks [65,66]. In mature CNS, microglia maintain the synaptic plasticity via secreting several soluble molecules that are accountable for controlling memory and learning and also for increasing LTP responses mediated by N-methyl-D-aspartate (NMDA). Microglia also mediate the basal glutamatergic signaling and regulation of GABAergic transmission via adenosine triphosphate (ATP) and brain derived neurotrophic factor (BDNF) [67,68]. BDNF is important for the phosphorylation of tyrosine kinase B, which is accountable for synaptic plasticity. Mouse models with microglia depletion exhibited decreased capacity in several learning tasks and reduced levels of motor learning linked with synaptic formation. Collectively, these findings suggest that microglia are crucial for synaptic remodeling and learning [64]. Microglia also play roles in activity-dependent structural remodeling driven via age-related factors and sensory input [60,69]. In Figure 2, we have summarized the beneficial effects of microglia in healthy adult brains.

## 4. Effect of Microglia on Aging Brain

Aging impairs the functions of tissues and cells because of the decreased level of cellular components and also due to the intracellular deposits of distorted organelles and macromolecules. In an aged brain, the immune system works in an incongruous pattern, which makes it more vulnerable to age-related dysfunctions and damages [70]. In the brain, these aforesaid events can further lead to microglial dystrophy, which is a sign of microglial senescence [71]. It has been reported that microglia become more reactive during aging [72,73]. During aging, microglia show an amoeboid-like structure and express elevated levels of cluster of differentiation 14 receptors, Toll-like receptors 4, and major histocompatibility complex class II antigens on their surface. In the healthy senile brain of aged mouse models, microglia were found to express increased levels of anti-(TGF-b, IL-10) and pro- (TNF-a, IL-1b, IL-6) inflammatory cytokines [73,74]. 

Aging seems to play a role as a priming stimulus to microglia like NDs. Aged microglia induce the release of an increased level of proinflammatory cytokines owing to any kind of infections, injury, and changes in the brain. Stimulation of microglia and weakened microglial response take place because of the age-linked alterations in microglial regulation [75]. Numerous studies have observed in aged individuals exposed to peripheral stimulation that an increased level of induced microglia is accountable for the elevated behavioral alterations including maladaptive sickness response [76]. In the aged brain, elevated cytokine release in response to altered immune response is also accountable for the cognitive deficit [77].

## 5. Role of Microglia in Alzheimer’s Disease

Microglia have a significant contribution in maintaining brain homeostasis, including maintenance of CNS integrity, providing protection to the CNS from pathogenic attacks, and surveying the whole brain parenchyma. Nonetheless, the homeostatic roles of microglia are lost in AD. Numerous findings have suggested that weakened or diseased microglia have significant contributions in AD pathogenesis. In Table 2, we have summarized both the protective and pathological roles of microglia in AD pathogenesis.

### 5.1. Microglial Mitophagy

It has been observed that induction of mitophagy may exert some beneficial effects in microglia including an ameliorated microglial function to phagocytose and suppression of neuroinflammation. In the brains of APP/PS1 mice, increased levels of mitophagy elevated the expressions of Aβ within microglia [94], which further indicates that elevated level of mitophagy can enhance or activate microglia-mediated phagocytosis and Aβ clearance in case of AD. These findings suggest that suppression of AD-associated pro-inflammatory responses, such as caspase-1 and NLRP3 can decrease the level of Aβ pathology [95]. Interestingly, microglia from NRLP3 and caspase-1 knock-out mouse models exhibit elevated phagocytotic function. Variations in mitophagy have been observed in the aging process and age-associated diseases [96,97]. In premature aging models, mitophagy markedly affected the survival and functions of neurons [98,99].

### 5.2. Role of Microglia in Amyloid Beta

Amyloid beta (Aβ) is derived from the amyloid precursor protein that plays a crucial role in AD pathogenesis [100]. Aβ plaques are formed because of the aberrant aggregation and accumulation of Aβ and these plaques are considered as one of the major pathological AD hallmarks [101]. Common Aβ subtypes including Aβ1–40 and Aβ1–42 also play crucial roles in AD development. Aβ oligomers have a significant contribution in early AD stages, whereas Aβ fibers have a significant contribution in prolonging the inflammatory response. It has been reported that activated microglia can generate an increased level of glutamic acid, which is induced via NMDA receptor through signaling mechanisms, that eventually can result in toxicity [102]. Outside the synapse, Aβ could be induced to elevate the deposition via the activated NMDA receptor [103]. Microglia possess various receptors on their surfaces that interact with Aβ and play role in chemotaxis to microglia, including receptor of advanced stage glycosylation end production and scavenger receptor (SR) [104]. It has been observed that macrophage colony-stimulating factor also plays role in chemotaxis, which is released via microglia and activated via Aβ. Various chemotactic factors including monocyte chemoattractant protein-1 (MCP-1) induce microglia to gather in Aβ deposition [105]. Activated microglia might involve themselves in Aβ phagocytosis via SRs and cause Aβ hydrolysis via the secretion of insulin hydrolytic enzymes, alpha secretases, and metalloproteinases [106].

### 5.3. Effects of Microglia in Tau Protein

NFTs are regarded as a major feature of AD pathogenesis. In normal conditions, tau interacts with tubulin and mediates microtubule stability and polymerization. Tau is a phosphorylated protein. When tau gets dissociated from microtubules in AD patients, it may become converted from the soluble form to the insoluble form, which can further lead to the formation of NFTs [107]. In nerve cells, an increased level of reactive microglia around tau has been reported in various animal models including P301Stau transgenic mouse models [108]. Furthermore, it has been revealed that the inflammation factor has the capacity to alter the function of related kinases, which can further result in tau phosphorylation [109]. In a study, Sy et al. [110] reported that the alteration of tau from a soluble form to an insoluble form in AD transgenic mice was linked with the inflammatory response and over-activity of glycogen synthase kinase-3 (GSK-3).

### 5.4. Effect of Microglia in Neuroinflammation

It is now well known that neuroinflammation has significant role in AD [111,112]. Furthermore, microglial activation precedes tau and Aβ pathologies within the brain of animal models and AD patients [113,114]. In AD brains, elevated concentrations of various inflammatory mediators including IL-1β have been repeatedly observed [115]. Inflammasomes are multi-protein complexes that have contributions in inflammation pathways in the cells. Following exposure of cells to danger-associated and pathogen-associated molecular patterns, microglia get activated and mediate Caspase-1 cleavage and secrete various inflammatory cytokines including IL-18 and IL-1β [116]. It has been observed that NLR Family Pyrin Domain Containing 1 (NLRP1) and NLRP3 are expressed in microglia and neurons in the brain [117]. Both NLRP1 and NLRP3 were found to be over-activated in the case of AD [118,119]. In microglia, Aβ can act as a strong activator of inflammasomes [119]. After microglia-mediated phagocytosis, Aβ triggers lysosomal injury and Cathepsin B leakage into the cytosol, which further results in activation of inflammasomes [119].

One of the major roles of microglia is responding against physical and immune-mediated injuries in the brain. In addition, microglia regulate the stress response against multiple pathological triggers in case of CNS disorders [120,121]. Without a cellular messenger, physiological responses towards infections in the periphery are mediated by microglia directly in the CNS [122], a response which markedly decreased after depletion of microglia [123]. It has been reported that the tendency of the brain to spread an inflammatory response is markedly elevated naturally with the aging process [124]. These triggers include ischemia or trauma-linked physical injury, cellular debris derived from neurodegeneration, protein aggregation (for instance amyloid plaques), CNS infections, and multiple sclerosis [125,126,127,128]. Furthermore, microglia possess various purinergic receptors that react to extracellular ADP and ATP, which are signs of potential cellular damage and necrosis [129]. After activation, physiological functions of microglia are changed, characterized via alterations in structure, along with elevated levels of cell surface receptors and increased expressions of cytokines and chemokines, all are reliant on the inducing events [130]. It has been confirmed by various studies that activation of microglia results in neurotoxic effects and disrupted synaptic activity, which can eventually result in cognitive deficits and neurodegeneration [131,132,133,134,135]. Nonetheless, in case of neuroinflammatory conditions, transient activation of microglia can be beneficial, since this activation can mediate the repairing and survival of neurons after brain damage via various anti-inflammatory signaling pathways [136,137].

### 5.5. Detrimental Activities of Microglia in AD

**Figure 3:**
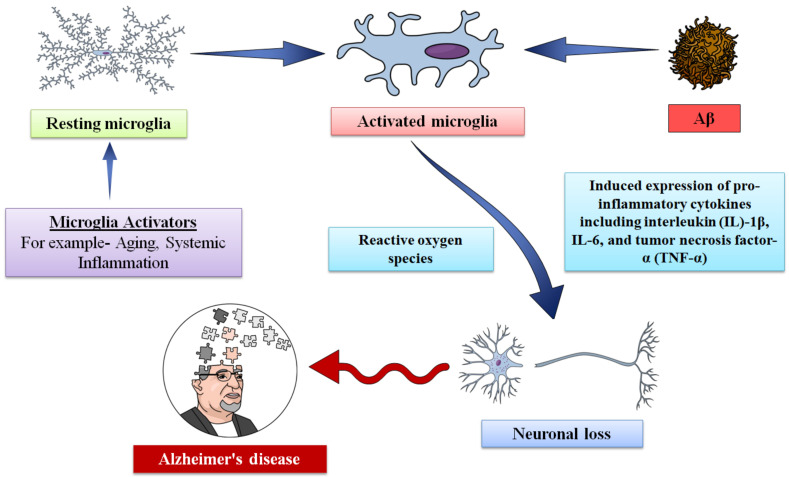
The detrimental roles of microglia in Alzheimer’s disease.

Even though various studies have revealed that appropriate microglial activity can provide protection against AD, however numerous studies have demonstrated that uncontrolled microglial function can be detrimental to neurons in the case of NDs. It has been reported that Aβ plaques appear a long time before clinical AD symptoms, however loss of synapses and tau pathology play roles in cognitive deficit during the progression of AD [138]. Microglia also release several toxic factors that can indirectly or directly damage neurons [17,139]. In Figure 3, we have summarized the detrimental roles of microglia in AD. 

## 6. Targeting Microglia for the Treatment of Alzheimer’s Disease

Numerous studies are ongoing to reverse or stop AD pathogenesis, however only a few concrete findings have resulted in the clinical treatment. Microglia play significant roles in health and diseases. On the other hand, dysfunctional microglia lose their phagocytic ability and consequently induce inflammatory pathways that worsen AD pathogenesis. Therefore, the development of therapies by targeting microglia might be a novel approach in AD treatment. 

### 6.1. Therapeutics to Modify Microglia

Microglia play dual roles in the progression of AD. It has been observed that early activations of microglia exert endogenous anti-inflammatory effects and neuroprotective activities via mediating Aβ clearance. Nonetheless, the load of Aβ increases with the advancement of AD. Furthermore, over-activated microglia obtain a pro-inflammatory phenotype, which further mediates the accumulation of Aβ and hastens AD pathogenesis. Thus, replenishing healthy microglia or removing dysfunctional microglia might have the potential to be used as novel AD therapies (Table 3). Various studies have confirmed that depleting microglia have significant effects in AD transgenic mice [140,141,142,143].

#### 6.1.1. CSF1R Inhibitors

The receptor of the colony-stimulating factor-1 (CSF1R) plays a crucial role in the development and survival of microglia, therefore chronic continuous administration of CSF1R inhibitors might be an effective and non-invasive method to selectively remove dysfunctional microglia. In mouse models, the number of microglial cells was decreased by around 70–80% after 3 months of administration of the selective CSF1R inhibitors including 5XFAD and PLX3397 [141]. Prolonged administration of PLX3397 in 5XFAD transgenic mouse models ameliorated cognitive deficit and amyloid pathology in the brain areas affected by AD [141]. It has been reported by an in vivo study that PLX3397 inhibited propagation of tau and triggered microglial depletion, which further resulted in neuroprotection [140]. PLX5622 (another CSF1R inhibitor) showed good brain-penetration activities and oral bioavailability. Moreover, chronic PLX5622 administration (until 4 or 7 months of age since 1.5 months of age) in 5XFAD mouse models resulted in the formation of Aβ plaques [142]. A reduced level of overall plaque load was also observed after blocking the CSF1R [143]. In 5XFAD transgenic mouse models (4 months old), ablation of microglia in Aβ plaque deposits during the progression of AD pathogenesis resulted in alteration of plaque structure from compact to diffuse. Interestingly, even during the peak period of Aβ plaque formation, microglia have a significant contribution in limiting the expansion of Aβ plaques.

Deletion of an enhancer of CSF1R, Fms intronic regulatory element, resulted in microglia-deficient animal models [169]. Under the control of the CX3CR1 (C-X3-C Motif Chemokine Receptor 1) promoter, Cre-induced recombination resulted in the selective expression of the diphtheria toxin receptor via microglia. Microglia were selectively removed by around 80% after the treatment with diphtheria toxin [170]. In AD, genetic interferences might provide a more instinctive method to explore immunological and physiological microglial functions. Even though ablation of microglia might be a novel option in AD treatment, various factors are needed to be evaluated and discussed, for instance accurate timing of microglial depletion and functional status of the microglia at certain AD stages. Therefore, key challenges must need to be dealt prior to the clinical availability of these therapies.

#### 6.1.2. Stem Cell Therapy

Replenishing healthy microglia might be beneficial in improving AD pathogenesis. Furthermore, stem cell transplantation has the therapeutic potential to repair the dysfunctional microglia in case of AD. Several microglia-like cells have already been derived from human stem cells, such as embryonic stem cells and induced pluripotent stem cells [162,163]. It has been observed that expression signatures of stem cell-derived microglial cells are similar to purified human fetal microglia and these stem cell-derived microglial cells respond rapidly to harmful stimuli and show effective phagocytosis [162,163,164]. After transplantation, stem cell-derived microglia have the capacity to survive and integrate into the brains of mouse models [92]. Multiple in vivo studies have confirmed the neuroprotective properties of transplanted stem cells. In transgenic mouse models of AD, stem cell therapies improved memory impairments and associated neuropathology [165,167,168].

### 6.2. Targeting Microglial Immunoreceptors

#### 6.2.1. Targeting TREM2 Gene

TREM2 and CD33 have been widely studied owing to their roles as crucial AD risk genes. It has been demonstrated that TREM2 is essential for the response of microglia to Aβ [89,171]. Various agonistic antibodies of TREM2, such as AL002a [157], AL002c [158], antibody 2 [156], and antibody 1 [156] exerted neuroprotective properties by elevating microglial responses to Aβ and via improving Aβ pathology. In a phase 1 clinical trial, clinical variants of AL002 and AL002c were found to be better tolerated [158]. It has been reported that reduction of proteolytic shedding can improve TREM2 function [159]. Interestingly, 4D9 (a monoclonal antibody) stabilized expressions of TREM2 on the cell surface via bivalent binding and decreased TREM2 shedding. Moreover, 4D9 increased in vitro and in vivo Aβ phagocytosis and ameliorated the microglial response to Aβ [159].

#### 6.2.2. Targeting CD33 Gene

CD33 gene polymorphisms are associated with AD pathogenesis and the domain for sialic acid-binding might be a potential target for CD33-mediated inhibition of Aβ phagocytosis [172]. Therefore, targeting the domain for sialic acid binding might be an auspicious strategy of AD treatment. P22 is a novel subtype-selective sialic acid mimetic [161]. In a CD33-dependent manner, P22 conjugated microparticles increased Aβ phagocytosis. In addition, CD33 inhibitory antibodies might also provide resistance to the neurotoxic properties of CD33. CD33 has also been identified as one of the potential targets for potential AD treatment [160]. On the other hand, multiple existing CD33 inhibitory antibodies might also be repurposed as therapies to treat AD. More studies are required to validate and evaluate the probability of utilizing antibodies for AD treatment.

### 6.3. Targeting Inflammatory Response Mediated by Microglia

#### 6.3.1. Non-Steroidal Anti-Inflammatory Drugs

Already a large number of studies have explored the use of non-steroidal anti-inflammatory drugs (NSAIDs) in AD treatment. Chronic administration of NSAIDs can improve AD pathogenesis. Ibuprofen (one of the most commonly used NSAIDs) has the ability to decrease the levels of proinflammatory cytokines, microglial activation, and Aβ plaque load in vivo and in vitro [151,173,174,175]. It has been observed that NSAIDs-mediated neuroprotective properties seem to be linked with peroxisome proliferator-activated receptor-gamma (PPARγ). After activation by NSAIDs, PPARγ can exert transcriptional regulation via suppressing the expressions of pro-inflammatory genes [174]. Therefore, pioglitazone (an agonist of PPARγ) was investigated in clinical AD research [150,152], but the related phase III trials were terminated because of its efficacy [176,177].

#### 6.3.2. NLRP3 Inflammasome Inhibitors

Activation of the microglial inflammasome (particularly NLRP3 (NOD-, LRR- and pyrin domain-containing protein 3) inflammasome) has the ability to induce AD pathogenesis. The NLRP3 inflammasome is a multiprotein complex that possesses the procaspase-1, adapter protein ASC, and NLRP3 protein. In AD, indirect or direct suppression of the NLRP3 inflammasome decreases the microglial inflammatory responses [178]. Various preclinical studies have also demonstrated the effectiveness of various inhibitors of NLRP3 inflammasome-targeting molecules [145,155,179]. Furthermore, the use of drugs targeting the NLRP3 inflammasome has also gained attention. Minocycline (an anti-inflammatory tetracycline) has the ability to cross the blood-brain barrier (BBB). Minocycline can decrease the levels of microglial activation and Aβ accumulation, probably via suppressing the NLRP3 inflammasome [146]. Various clinical studies have assessed the neuroprotective properties of minocycline in AD treatment. Unfortunately, targeting the inflammatory response via minocycline could not delay the advancement of cognitive deficits in AD patients [180]. Edaravone is commonly utilized to treat cerebral infarction and it also plays a role as a scavenger of free radicals. Growing evidence has demonstrated the importance of the anti-inflammatory effects of edaravone on Aβ-induced microglial activation via suppression of NLRP3 inflammasome activation [181]. Moreover, numerous in vitro and in vivo studies have demonstrated the neuroprotective properties of edaravone [147,153,154,181]. Since edaravone can effectively penetrate BBB, therefore it has the potential to be an effective therapeutic agent for the treatment of AD.

#### 6.3.3. P2X7R Inhibitors

Purinergic P2X receptor 7 (P2X7R) is a member of the purinergic receptor. P2X7R acts as a strong NLRP3 inflammasome activator and plays role in facilitating the secretion of pro-inflammatory mediators. Thus, antagonizing P2X7R might alleviate the AD-related microglial inflammatory responses [144,178]. The expression of P2X7R was found to be colocalized with Aβ plaque-linked microglia. In addition, an increased level of microglial P2X7R expression was detected in the brains of AD patients as compared to controls [148]. These results also have been demonstrated in AD transgenic mouse models [182] and in in vitro microglia cultures [148]. Oxidized ATP is a selective P2X7R inhibitor that can counter microglial responses triggered via co-stimulation with selective agonists of P2X7R and Aβ1–42 [148]. Brilliant blue G (another inhibitor of P2X7R) reduced microgliosis and antagonized the inflammatory responses exerted by a P2X7R agonist in an AD rodent model [149]. Nimodipine (a calcium channel blocker) showed neuroprotective properties by suppressing the secretion of mature IL-1β in Aβ-induced microglia and decreasing the levels of activated Nuclear factor-kappa B [144]. Even though antagonists of P2X7R have exhibited promising effects in animal and cellular research, however such antagonists have not been incorporated into clinical studies.

## 7. Future Directions

Microglia have long been assumed to have contributed in AD owing to their capacity to respond to neuronal dysfunctions including tau and Aβ aggregates [183]. Since microglia have capacities to react and sense their environment, therefore reactive microglia may have a significant contribution at the early stages of AD progression and might result in the detection of early AD biomarkers. Since microglia can crosstalk with non-neuronal immune cells and cause functional alterations in astrocytes [184], therefore microglia can be a potential drug target to limit or stop the progression of AD. Still, the precise roles of various subtypes of reactive microglia in the case of AD are not clear and require more studies. Multiple technological breakthroughs are now allowing researchers to explore the roles of microglia in AD. Better knowledge regarding the roles of microglia in AD initiation and advancement is estimated to renovate the interest of big pharmaceutical companies to re-invest in this research field and development of novel anti-AD drug discoveries.

Multiple factors including communication with the periphery, health status, molecular diversity, species, age, and sex need to be taken into consideration while evaluating the role of microglial cells in AD. Indeed, advances in the areas of nanotechnology have empowered the development of nanotherapeutic platforms that may overcome the challenges of targeted drug delivery to the CNS. Without compromising stability, certain active therapeutic agents for regulation microglial activation pathways and for precise suppression of toxic protein aggregations can also be combined in the nanoparticle structure. Therefore, microglia-targeted nanotherapeutic particles and nanodrugs might be able to tackle several pathological AD determinants and to mediate the shift of microglial phenotype spectrum towards a more neuroprotective condition [185]. Considering all these factors can be challenging, however this approach may lead to the development of novel therapeutic approaches and decrease the AD-linked socio-economic burden [186].

## 8. Conclusions

Currently available therapies for AD provide symptomatic treatment only, instead of targeting the underlying mechanisms associated with AD. Therefore, there is a strong need for treatment options that can interact with the mechanisms of AD pathogenesis and slow down its advancement. Neuroinflammation is one such downstream target, which is a cause instead of a consequence of neurodegeneration. Mechanisms that are linked with AD pathogenesis are highly complex and microglia are the key neuroinflammation modulators. In addition, microglia have a significant contribution in triggering synaptic dysfunction and loss; however, the precise and exact mechanisms are yet to be fully revealed. Thus, better knowledge regarding the molecular and cellular mechanisms of the microglia–synapse interaction is required on an urgent basis for the development of novel anti-AD therapies. Moreover, more studies are required regarding whether or not the prevention of microglia-mediated removal of synapses decreases cognitive deficits and averts neurodegeneration.

## Figures and Tables

**Table 2:** The protective and pathological roles of microglia in AD pathogenesis [78].

Event	Mediator	Effect on Microglial Function	References
Microglial mitophagy	High mobility group box 1/receptor for advanced glycation endproducts signaling mechanisms	Significant blockage of late-stage mitophagy in microglia	[79]
Role of microglia in amyloid beta (Aβ)	*Apolipoprotein E Gene*	*Apolipoprotein E ε4* genotype is related to diminished Aβ plaques	[80]
	Receptor for advanced glycation end products	Exerts dual effects in Aβ phagocytosis	[81]
	Scavenger receptor class A	Mediates microglial adhesion to Aβ and elevates the level of Aβ uptake by microglia	[82]
	Class B scavenger receptor	Exerts dual effects in Aβ phagocytosis	[83]
	*Triggering receptor expressed on myeloid cells 2 Gene*	Exerts dual effects in Aβ phagocytosis	[84]
	*Complement C3b/C4b Receptor 1 Gene*	Mediates microglia-mediated Aβ phagocytosis	[85]
	*CD33* Gene	Decreases microglia-mediated Aβ phagocytosis	[31]
	ATP Binding Cassette Subfamily A Member 7 Gene	Mediates microglia-mediated Aβ phagocytosis	[86]
Role of microglia in neuroinflammation	*C-X3-C Motif Chemokine Receptor 1*	Deficiency of this inflammatory adipose chemokine system deteriorates tau phosphorylation	[87]
	NOD-like receptor family pyrin domain-containing 3	Exacerbates inflammatory response mediated by microglia	[88]
	Suppressors of cytokine signaling	Shows protective properties by balancing the level of inflammatory response	[89]
Role of microglia in tau pathology	*Triggering receptor expressed on myeloid cells 2 Gene*	Mediates intraneuronal tau aggregation	[90]
	*Apolipoprotein E Gene*	*Apolipoprotein ε4* genotype significantly worsens neurodegeneration mediated by tau	[91]
	Colony-stimulating factor 1 receptor	Suppression of colony-stimulating factor 1 receptor results in the reduction of tau-mediated neurodegeneration	[92,93]

**Table 3:** Microglial drug targets in Alzheimer’s disease treatment [78].

Therapeutic Approaches	Therapeutics	Mechanisms	References
Therapies targeting inflammatory response in microglia	Nimodipine, edaravone, minocycline, JC-124, MCC950, pioglitazone, ibuprofen	Amelioration of over-activated microglia and suppression of microglia-linked inflammatory responses	[104,144,145,146,147,148,149,150,151,152,153,154,155]
Therapies targeting microglial immunoreceptors	AL002c, AL002a, AL002, monoclonal antibody 4D9	Improvement of TREM2 function to elevate microglial reactions towards Aβ	[156,157,158,159]
	Lintuzumab, P22	Suppression of CD33 function to elevate the level of Aβ phagocytosis	[160,161]
Microglia modifying therapies	Inhibitors of colony-stimulating factor 1 receptor: PLX5622, PLX3397	Reducing dysfunctional microglia	[141,142]
	Stem cell therapy	Resupplying healthy microglia	[92,162,163,164,165,166,167,168]
